# Profiling physical activity motivation based on self-determination theory: a cluster analysis approach

**DOI:** 10.1186/s40359-015-0059-2

**Published:** 2015-01-20

**Authors:** Stijn AH Friederichs, Catherine Bolman, Anke Oenema, Lilian Lechner

**Affiliations:** Faculty of Psychology and Educational Sciences, Open University of the Netherlands, P.O. Box 2960, 6401 DL Heerlen, The Netherlands; Department of Health Promotion, Maastricht University, P.O. Box 616, 6200 MD Maastricht, The Netherlands

**Keywords:** Physical activity, Self-determination theory, Motivational profile

## Abstract

**Background:**

In order to promote physical activity uptake and maintenance in individuals who do not comply with physical activity guidelines, it is important to increase our understanding of physical activity motivation among this group. The present study aimed to examine motivational profiles in a large sample of adults who do not comply with physical activity guidelines.

**Methods:**

The sample for this study consisted of 2473 individuals (31.4% male; age 44.6 ± 12.9). In order to generate motivational profiles based on motivational regulation, a cluster analysis was conducted. One-way analyses of variance were then used to compare the clusters in terms of demographics, physical activity level, motivation to be active and subjective experience while being active.

**Results:**

Three motivational clusters were derived based on motivational regulation scores: a low motivation cluster, a controlled motivation cluster and an autonomous motivation cluster. These clusters differed significantly from each other with respect to physical activity behavior, motivation to be active and subjective experience while being active. Overall, the autonomous motivation cluster displayed more favorable characteristics compared to the other two clusters.

**Conclusions:**

The results of this study provide additional support for the importance of autonomous motivation in the context of physical activity behavior. The three derived clusters may be relevant in the context of physical activity interventions as individuals within the different clusters might benefit most from different intervention approaches. In addition, this study shows that cluster analysis is a useful method for differentiating between motivational profiles in large groups of individuals who do not comply with physical activity guidelines.

## Background

Regular physical activity (PA) has been shown to be highly beneficial for health, and to decrease the risk of many adverse health conditions such as coronary heart disease, type 2 diabetes and breast and colon cancer (Lee et al. 2012). Because of these beneficial effects, international PA guidelines state that for enhanced health, adults should accumulate 30 min or more of moderate intensity PA for at least 5 days per week (Garber et al. 2011). Unfortunately, many adults worldwide do not comply with these guidelines (Hallal et al. 2012). This is also the case in the Netherlands: in 2011 almost half of Dutch adults were insufficiently active according to these guidelines (Hildebrandt et al. 2013). Therefore, promoting PA behavior at the (inter)national population level is a public health priority (Heath et al. 2012; Glasgow & Emmons 2007). In order to promote PA uptake and maintenance in individuals who do not comply with PA guidelines, a proper understanding of the determinants of PA in this subgroup is needed (Hall et al. 2010; Bauman et al. 2012). One determinant thought contributing substantially to PA uptake is the *motivation* to become physically active (Bauman et al. 2012; Duncan et al. 2010). Hence, it is important to increase our understanding of PA motivation among individuals who do not comply with the PA guidelines (Hall et al. 2010). Self-determination theory (SDT) offers a theoretical framework for understanding motivation (Ryan & Deci 2000; Deci & Ryan 2008) and the literature indicates that SDT can be especially helpful for understanding PA motivation (Teixeira, Carraca et al. 2012).

One of the main principles of SDT is that motivation varies in the extent to which it is experienced as autonomous or controlled (Ryan & Deci 2000; Deci & Ryan 2008). SDT proposes several forms of motivation which lie on a continuum from the most controlled form to the most autonomous (in which the perceived locus of causality is fully internal). The least autonomous, most controlled form of motivation of the continuum is external regulation which comprises satisfying an external demand which can be either physically or symbolically present in the social environment. In this form of motivation, an individual acts in line with this demand, in order to avoid punishment, or to receive an external reward and the perceived locus of causality is fully external. In introjected regulation one internalizes the behavior regulation a little more. Introjected regulation occurs when one is driven by internal pressures, which can be either feelings of guilt or shame when the behavior is not performed, or positive self-views when the behavior is performed. Identified regulation entails a largely internal perceived locus of causality. This form of motivation involves being driven by the pursuit of specific, personally important outcomes of the behavior. In integrated regulation, an individual has fully integrated motivation within him or herself and acts because a behavior is congruent with personal beliefs and values. The most autonomous form of motivation is intrinsic motivation. This form of motivation occurs when someone is driven by interest in or enjoyment with the task itself. Motivation comes completely from “within” for intrinsic motivation. Finally, amotivation describes a lack of any intention to engage in a behavior (Ryan & Deci 2000; Deci & Ryan 2008).

By defining these different forms of motivation, SDT accounts for the *quality* of motivation rather than its *quantity* (Ryan & Deci 2000; Deci & Ryan 2008). Activities that are mainly driven by controlled forms of motivation (external regulation and introjected regulation) are hypothesized to generate intrapersonal conflict which hinders the availability of volitional resources such as the capacity to exert sustained effort (Koestner et al. 2008). Although controlled motivation may sometimes motivate short-term behavior, it is expected not to be capable of sustaining maintenance over longer periods of time (Ryan & Deci 2000; Deci & Ryan 2008; Teixeira, Carraca et al. 2012; Markland & Ingledew 2007). Individuals who are autonomously motivated to be active, often display more positive emotions, higher levels of perceived behavioral competence and reflective self-endorsement, and are typically more willing to engage in the behavior for prolonged periods of time (Teixeira, Carraca et al. 2012). Therefore, these individuals are usually more likely to engage in long-term maintenance than those who are merely driven by controlling motives (Ryan & Deci 2000; Deci & Ryan 2008; Teixeira, Carraca et al. 2012; Markland & Ingledew 2007).

In the present literature on PA and PA promotion, much attention is given to SDT and the quality of motivation (Teixeira, Carraca et al. 2012; Markland & Ingledew 2007). However, the majority of these studies employed a variable-centered approach, by evaluating the effects that each of the motivational regulations exerts on outcomes using regression analyses or structural equation modeling (Guerin & Fortier 2012; Stephan et al. 2010; Matsumoto & Takenaka 2004). While these strategies are technically correct, they do not take into account the different motivational configurations that may be present in different people. This represents a shortcoming in SDT research, as motivation is a dynamic construct, and it is common for individuals to report a combination of multiple motivational regulations for a given domain at the same time (Deci & Ryan 2002; Vallerand 1997; Patrick 2014). Analyzing these data (exclusively) using a variable-centered approach, leads to a loss of relevant information on how different regulations operate together within an individual.

One way to better account for individual motivational configurations, and their influence on outcomes, is to use a person-centered approach (Pintrich 2003; Ratelle et al. 2007; Vansteenkiste et al. 2009). Several authors have recommended using this approach by assessing how different types of motivation are combined to form motivational profiles. A motivational profile reflects a specific combination of motivation scores which is likely to provide more information compared to an individual’s scores on the separate motivational regulations (Vansteenkiste et al. 2009).

As described above, the person-centered approach is theoretically advantageous as it increases our understanding of how different motivational regulations co-exist in individuals. From a practical point of view, the person-centered approach is also helpful as it could lead to better tailoring PA interventions for particular groups (Guerin & Fortier 2012; Vansteenkiste et al. 2009). For example, within the context of a PA intervention, a group of individuals with moderate intrinsic motivation, high identified regulation and low introjected/external regulation might benefit most from an intervention that focuses on strengthening existing motivation and forming new challenging action plans. A group of individuals characterized by low intrinsic motivation, low identified regulation and high introjected/external regulation might benefit most from an intervention that focuses on internalizing the perceived locus of causality, by evoking more autonomous motivation, for instance through the use of a value-based approach (Miller & Rollnick 2013).

Recently, PA researchers have begun to study motivational profiles based on SDT using cluster analysis. Cluster analysis is a statistical method that groups individuals into clusters based on similar characteristics (Hair & Black 2000; Hair et al. 1998). Until now, most cluster studies on SDT and PA have focused on (junior) athletes (Gillet et al. 2013; Gillet et al. 2009; Murcia et al. 2007; Caglar & Asci 2010; Vlachopoulos et al. 2000), physical education (Ntoumanis 2002; Boiché et al. 2008) and elderly individuals (Stephan et al. 2010; Ferrand et al. 2014). Overall, these studies show that clustering has advantages over and above categorizing individuals as low or high in autonomous motivation, since it indeed provides more information about how different regulations together influence behavior.

Only a few studies have assessed motivational profiles regarding PA in adult populations (Guerin & Fortier 2012; Matsumoto & Takenaka 2004). One of these studies investigated predominantly active individuals and found four clusters: a self-determined motivation cluster, a moderate motivation cluster, a non-self-determined motivation profile and an amotivation cluster (Matsumoto & Takenaka 2004). The results further showed that individuals from the self-determined motivation cluster were more frequently in the maintenance stage of behavior change than members from the other clusters (Matsumoto & Takenaka 2004). As pointed out above, it is important to specifically investigate PA motivation in individuals who do not comply with the PA guidelines, because these individuals can achieve the greatest health benefits by becoming more physically active (Hall et al. 2010). Gaining more insight into motivational profiles in this specific population is thus essential. However, to our knowledge, only one study has assessed motivational profiles in this group finding three clusters: a self-determined cluster, a motivated cluster and a low motivation cluster (Guerin & Fortier 2012). The authors found that individuals from the self-determined and the motivated cluster displayed higher levels of enjoyment than those from the low motivation cluster. Unfortunately, enjoyment was the only variable measured in this study, and the sample size was rather limited (n = 120).

In short, there is hardly any literature on motivational profiles in individuals who do not comply with PA guidelines even though his is a highly relevant population for such a study. Therefore, with the present study, we aimed to assess motivational profiles in a large sample of adults who do not comply with PA guidelines. The first objective of this study was to identify (and describe) motivational profiles by conducting cluster analysis on motivational regulation scores. Secondly, we aimed to compare the derived clusters in terms of individual characteristics. By assessing several relevant self-report measures, we aimed to obtain a clear view of the characteristics of the profiles (and the differences between these profiles). In addition to demographics, PA level, intention and commitment we also intended to compare the clusters in terms of subjective experience while being active, such as the extent to which one perceives being active as stressful or enjoyable, and the degree to which one feels competent while being active (McAuley et al. 1989).

While the present study is mostly exploratory in nature, we still attempted to prepare hypotheses concerning the expected number of clusters, and the nature of these clusters. We reviewed the literature for studies that assessed individual motivational profiles in the context of work and organization (Moran et al. 2012; Van den Broeck et al. 2013), education (Ratelle et al. 2007; Vansteenkiste et al. 2009; Hayenga & Corpus 2010; Liu et al. 2009; Wormington et al. 2012; Corpus & Wormington 2014) and PA (Guerin & Fortier 2012; Stephan et al. 2010; Matsumoto & Takenaka 2004; Gillet et al. 2013; Gillet et al. 2009; Murcia et al. 2007; Caglar & Asci 2010; Vlachopoulos et al. 2000; Ntoumanis 2002; Boiché et al. 2008; Ferrand et al. 2014). Some of these studies found two (Vlachopoulos et al. 2000; Ferrand et al. 2014) or five (Moran et al. 2012) clusters. In most studies, however, cluster solutions of three (Guerin & Fortier 2012; Stephan et al. 2010; Ratelle et al. 2007; Gillet et al. 2013; Murcia et al. 2007; Ntoumanis 2002; Boiché et al. 2008; Corpus & Wormington 2014) or four (Matsumoto & Takenaka 2004; Vansteenkiste et al. 2009; Gillet et al. 2009; Caglar & Asci 2010; Van den Broeck et al. 2013; Hayenga & Corpus 2010; Liu et al. 2009; Wormington et al. 2012) clusters were found. Among the PA studies, three-cluster solutions were found most often (Guerin & Fortier 2012; Stephan et al. 2010; Gillet et al. 2013; Murcia et al. 2007; Ntoumanis 2002; Boiché et al. 2008). In more than half of the studies we consulted, two opposite clusters were found: one cluster with high levels of autonomous motivation and low levels of controlled motivation, and one cluster with low levels of autonomous motivation and high levels of controlled motivation (Vansteenkiste et al. 2009; Murcia et al. 2007; Ntoumanis 2002; Boiché et al. 2008; Moran et al. 2012; Van den Broeck et al. 2013; Hayenga & Corpus 2010; Liu et al. 2009; Wormington et al. 2012; Corpus & Wormington 2014). Furthermore, several studies found a cluster with high levels of both autonomous and controlled motivation (Stephan et al. 2010; Ratelle et al. 2007; Vansteenkiste et al. 2009; Gillet et al. 2013; Gillet et al. 2009; Caglar & Asci 2010; Moran et al. 2012; Van den Broeck et al. 2013; Hayenga & Corpus 2010; Liu et al. 2009; Wormington et al. 2012) or low levels of both types of motivation (Matsumoto & Takenaka 2004; Vansteenkiste et al. 2009; Van den Broeck et al. 2013; Hayenga & Corpus 2010; Liu et al. 2009; Wormington et al. 2012; Corpus & Wormington 2014). Based on these studies, we expected to find three or four clusters. We further anticipated to find one cluster with high levels of autonomous motivation and low levels of controlled motivation, and one cluster with low levels of autonomous motivation and high levels of controlled motivation, as this combination was often found in earlier studies (Vansteenkiste et al. 2009; Murcia et al. 2007; Ntoumanis 2002; Boiché et al. 2008; Moran et al. 2012; Van den Broeck et al. 2013; Hayenga & Corpus 2010; Liu et al. 2009; Wormington et al. 2012; Corpus & Wormington 2014). In addition to these two clusters, we also expected a cluster that is characterized by either high or low levels on both types of motivation. As previously mentioned, it is assumed that autonomous forms of motivation are often accompanied by positive emotions, perceptions of behavioral competence and higher levels of reflective self-endorsement (Teixeira, Carraca et al. 2012). Therefore, we expected that clusters characterized by high levels of autonomous motivation would display the most favorable outcomes on PA behavior and PA related psychological constructs.

## Methods

For this study, the baseline data from the *I Move* trial (Friederichs et al. 2014) was used. In this trial, the effectiveness of a novel web-based PA intervention was tested. The *I Move* trial was approved by the Medical Ethics Committee of Atrium–Orbis–Zuyd and was registered with the Dutch Trial Register (NTR4129). The present study focused on the data from the baseline questionnaire filled out by participants before being allocated to the web-based PA intervention.

Participants for the *I Move* study were recruited via advertisements in national newspapers, social media, and an online panel. Participants were eligible for participation in this trial if they were between 18 and 70 years old, did not have a condition that seriously affected their ability to be physically active, did not participate in one of the *I Move* pilot studies, and were less physically active than 5 days per week for 60 minutes per day (Friederichs et al. 2014). All eligible individuals agreeing to participate were are asked to sign an online informed consent form.

In total, 8,585 individuals clicked on the *‘I want to participate’* button on the *I Move* study website; 4,302 individuals passed the inclusion criteria and gained access to the baseline questionnaire. Finally, 3,165 individuals completed the baseline questionnaire (31.1% male; age 45.0 ± 12.9). Since the current study aimed to focus on a relatively sedentary population, those individuals who reported being physically active on at least five days per week for at least 30 minutes per day were excluded from this study (n = 607). According to the guidelines of the SQUASH (the PA questionnaire used in this study) individuals who reported spending more than 6,720 minutes on PA per week were also excluded (n = 32). In addition, 53 univariate and multivariate outliers were removed (this is discussed in the statistical analysis section). The resulting sample for the present study consisted of 2,473 individuals (31.6% male; age 44.6 ± 12.9; 74.2% living together or married; 60.2% highly educated; BMI 26.2 ± 5.0; weekly days with ≥ 30 minutes moderate to vigorous PA 2.5 ± 1.2).

### Measures

#### Demographics

Age, gender, weight, height, relational status and highest completed educational level were assessed. Educational level was categorized into high (higher vocational school or university level) and low (elementary education, medium general secondary education, preparatory vocational school, lower vocational school, higher general secondary education, preparatory academic education, medium vocational school), according to the Dutch educational system.

#### Motivational regulation

Motivational regulation towards PA was assessed using the Exercise Self-Regulation Questionnaire (SRQ-E). The SRQ-E contained the subscales external regulation, introjected regulation, identified regulation, and intrinsic motivation (Ryan & Connell 1989). These concepts and their Cronbach’s alphas (based on the data from this study) are described in Table 1.

**Table 1:** **Description of the assessed variables**

**Concept**	**Questionnaire**	**# items**	**Example question**	***α***
External regulation	Exercise Self-Regulation Questionnaire (SRQ-E)	4	I try to be sufficiently physically active because others would be angry at me if I did not.	.73
			*Totally disagree (1) - Totally agree (7)*	
Introjected regulation	Exercise Self-Regulation Questionnaire (SRQ-E)	4	I try to be sufficiently physically active because I feel guilty if I do not exercise regularly.	.73
			*Totally disagree (1) - Totally agree (7)*	
Identified regulation	Exercise Self-Regulation Questionnaire (SRQ-E)	4	I try to be sufficiently physically active because exercising helps me feel better.	.88
			*Totally disagree (1) - Totally agree (7)*	
Intrinsic motivation	Exercise Self-Regulation Questionnaire (SRQ-E)	4	I try to be sufficiently physically active because it’s fun.	.88
			*Totally disagree (1) - Totally agree (7)*	
Interest/enjoyment	Intrinsic Motivation Inventory (IMI)	7	I enjoy being physically active.	.92
			*Totally disagree (1) - Totally agree (7)*	
Perceived competence	Intrinsic Motivation Inventory (IMI)	6	I think I am pretty good at physical activities.	.87
			*Totally disagree (1) - Totally agree (7)*	
Effort/importance	Intrinsic Motivation Inventory (IMI)	5	I put a lot of effort into physical activities.	.82
			*Totally disagree (1) - Totally agree (7)*	
Pressure / tension	Intrinsic Motivation Inventory (IMI)	5	During physical activities, I am very tense	.82
			*Totally disagree (1) - Totally agree (7)*	
Perceived choice	Intrinsic Motivation Inventory (IMI)	7	I feel that it is my own choice to perform physical activities.	.81
			*Totally disagree (1) - Totally agree (7)*	
Value/usefulness	Intrinsic Motivation Inventory (IMI)	7	I believe being physically active could be valuable to me.	.88
			*Totally disagree (1) - Totally agree (7)*	
Intention	(Sheeran & Orbell 1999)	3	To what extent are you planning to be sufficiently physically active?	.93
			*Not at all (1) - Very much (10)*	
Commitment	(Webb & Sheeran 2005)	3	How committed are you to being physically active?	.82
			*Not at all (1) - Very much (5)*	

#### PA level

Total weekly days of sufficient PA and minutes of moderate to vigorous PA were assessed using the validated self- administered Dutch Short Questionnaire to Assess Health Enhancing Physical Activity (SQUASH) (Wendel-Vos et al. 2003).

Total weekly minutes of moderate to vigorous PA (MVPA) was computed by multiplying the frequency (how many days per week), and duration (how many hours and minutes per day) of leisure and transport walking, leisure and transport cycling, sports, gardening, household chores and odd jobs performed with moderate or vigorous intensity. The reproducibility (*r*_spearman_ = 0.58; 95% CI = 0.36–0.74) and relative validity (*r*_spearman_ = 0.45; 95% CI = 0.17–0.66) of the SQUASH are reasonable for the general adult population (Wendel-Vos et al. 2003).

Total weekly days of sufficient PA was measured by a single item: “How many days per week are you, in total, moderately physically active by undertaking, for example, brisk walking, cycling, chores, gardening, sports, or other physical activities for at least 30 minutes?”. Prior research provided support for the validity and reliability of single-item self-reports of PA (Milton et al. 2011; Milton et al. 2013) and several studies found the single item PA measure to be among the most accurate of PA questionnaires, when compared to accelerometer output (Wanner et al. 2013; van Poppel et al. 2010).

#### Other PA related measures

The Intrinsic Motivation Inventory (IMI) was used to assess the feelings that participants experience while being physically active, referred to as ‘subjective experience’ in the remainder of the paper. The IMI encompasses the following subscales: interest/enjoyment, perceived competence, effort/importance, pressure/tension, perceived choice and value/usefulness (McAuley et al. 1989). In addition, intention (Sheeran & Orbell 1999) and commitment (Webb & Sheeran 2005) were assessed. These concepts and their Cronbach’s alphas (based on the data from this study) are described in Table 1.

### Statistical analyses

All analyses were conducted using SPSS for Windows (Version 22). In order to generate motivational profiles based on the motivational regulation scores, a cluster analysis was conducted. The analysis was conducted in two steps, using a combination of hierarchical and nonhierarchical clustering approaches, as recommended by several authors (Hair et al. 1998; Gore 2000; Tan et al. 2006) since it allows researchers to form clusters with high internal and external homogeneities (Hair & Black 2000). Prior to conducting the cluster analysis, the motivational regulation scores were transformed into z-scores. Since hierarchical cluster analyses are sensitive to outliers, multivariate outliers (individuals with Mahalanobis Distance > 18.47, *p* < .001) and univariate outliers (individuals with motivational regulation scores of more than 3 SD below or above the mean) were removed from the dataset. The hierarchical cluster analysis was conducted using Ward’s method based on squared Euclidian distances. Ward’s method was used because it trivializes the within-cluster differences that are found in other methods (Aldenderfer & Blashfield 1984). The extracted initial cluster centers were then used as non-random starting points in an iterative k-means clustering procedure. The number of clusters was derived from the agglomeration schedule, by locating the largest increase in coefficients (Hair & Black 2000; Hair et al. 1998).

In order to examine the stability of the cluster solutions we used a double-split cross-validation procedure (Vansteenkiste et al. 2009). The sample was randomly split into halves (subsample A and B) and the two-step cluster procedure was applied to each half. After that, the participants of subsample A were assigned to new clusters using an iterative k-means cluster procedure based on the cluster centers of subsample B and vice versa. The new cluster solutions were then compared for agreement with the original cluster solutions in both subsamples using Cohen’s kappa, in which a kappa of at least 0.60 was considered acceptable (Vansteenkiste et al. 2009). Finally, the cluster centers from the subsample with the highest Cohen’s kappa were used to create the definitive cluster solution in the combined dataset, using an iterative k-means clustering procedure.

Between-cluster differences regarding demographic variables were assessed using analyses of variance (ANOVA) and Chi-square tests. Between-cluster differences in terms of (1) motivational regulation and (2) subjective experience while being active and intention/commitment towards PA were assessed using two multivariate analysis of variance (MANOVA), followed by ANOVAs if Pillai’s trace was significant. Differences between the clusters with regard to PA behavior were assessed using ANOVAs. For all the ANOVAs that were significant, Bonferroni post-hoc tests were performed. All analyses were conducted using a significance level of .05.

## Results

**Figure 1:**
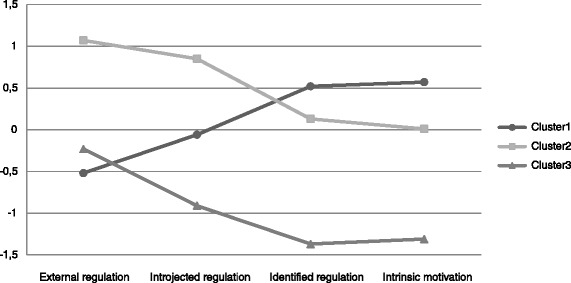
**Motivational regulation z-scores among clusters.**

The double-split cross-validation procedure resulted in a Cohen’s kappa’s of .872 (subsample A) and .999 (subsample B). The final cluster solution consisted of three clusters (see Figure 1). The results of the MANOVA implied significant group differences on the motivational regulation scores (Pillai’s trace 1.238; *p* < .001). Univariate testing indicated all between cluster differences were significant. The z-scores and raw scores of the four motivational regulations are reported in Table 2.

**Table 2:** **Mean z-scores and raw scores of motivational regulation per cluster**

	**Cluster 1:**		**Cluster 2:**		**Cluster 3:**		***F*** **(df = 2)**
	***Autonomous motivation***		***Controlled motivation***		***Low motivation***		
	**n = 1310**		**n = 610**		**n = 553**		
	**z-scores**	**raw scores**	**z-scores**	**raw scores**	**z-scores**	**raw scores**	
External regulation	−0.52 ± 0.39^a^	1.23 ± 0.37^a^	1.07 ± 0.79^b^	2.75 ± 0.76^b^	−0.23 ± 0.70^c^	1.50 ± 0.67^c^	1605.05***
Introjected regulation	−0.06 ± 0.86^a^	3.00 ± 1.07^a^	0.85 ± 0.70^b^	4.15 ± 0.88^b^	−0.91 ± 0.61^c^	1.93 ± 0.77^c^	765.72***
Identified regulation	0.52 ± 0.54^a^	5.81 ± 0.74^a^	0.13 ± 0.65^b^	5.28 ± 0.90^b^	−1.37 ± 0.89^c^	3.23 ± 1.22^c^	1626.20***
Intrinsic motivation	0.57 ± 0.63^a^	5.39 ± 0.96^a^	0.01 ± 0.70^b^	4.55 ± 1.06^b^	−1.31 ± 0.67^c^	2.54 ± 1.02^c^	1638.94***

For each variable, means with different superscripts indicate a significant difference at P < .05 using Bonferroni post-hoc tests.

***P < .001.

According to the criterions as proposed by Hodge et al. z-scores below −0.5 were classified as low, z-scores between −0.5 and 0.5 as moderate, and z-scores above 0.5 as high (Hodge & Petlichkoff 2000). The first cluster, labeled the *autonomous motivation* cluster, comprised 52.9% (n = 1310) of individuals. Members of this cluster scored high on identified regulation and intrinsic motivation, moderate on introjected regulation and low on external regulation. The second cluster (24.7%; n = 610) was labeled the *controlled motivation* cluster as individuals in this cluster scored high on introjected and external regulation, and moderate on identified regulation and intrinsic motivation. The third and smallest cluster (22.4%; n = 553) was labeled the *low motivation* cluster. Members of this cluster scored moderate on external regulation, and low on all the other motivational regulations.

### Differences between clusters: demographics

As seen in Table 3, individuals from the *autonomous motivation* cluster were on average more highly educated than those from the other two clusters. In addition, the average BMI in the autonomous motivation cluster was lower than in the other subgroups.

**Table 3:** **Means of PA behavior, intention, commitment and the IMI scales per cluster**

	**Cluster 1:**	**Cluster 2:**	**Cluster 3:**	***F/Chi^2*** **(df = 2)**
	***Autonomous motivation***	***Controlled motivation***	***Low motivation***	
	**n = 1310**	**n = 610**	**n = 553**	
Gender: % male	30.7%	32.6%	31.8%	0.80
Age	45.2 ± 12.7	43.9 ± 13.6	44.0 ± 12.6	2.91
Marital status: % married/living together	74.7%	73.3%	74.0%	0.79
Education: % high education	63.0%	56.1%	58.2%	9.47**
BMI	25.5 ± 4.4^a^	26.9 ± 5.6^b^	27.0 ± 5.4^b^	25.04***
Interest/enjoyment	5.73 ± 1.00^a^	4.85 ± 1.12^b^	3.91 ± 1.31^c^	549.64***
Perceived competence	4.78 ± 1.09^a^	4.08 ± 1.08^b^	3.58 ± 1.17^c^	254.52***
Effort/importance	5.42 ± 0.99^a^	4.72 ± 0.99^b^	3.99 ± 1.13^c^	395.69***
Pressure/tension	1.75 ± 0.81^a^	2.62 ± 1.10^b^	2.33 ± 1.15^c^	188.30***
Perceived choice	5.93 ± 0.89^a^	4.84 ± 1.02^b^	4.98 ± 1.12^c^	345.12***
Value/usefulness	6.47 ± 0.49^a^	6.00 ± 0.71^b^	5.54 ± 0.97^c^	384.51***
Intention	7.77 ± 1.51^a^	6.86 ± 1.50^b^	5.35 ± 1.84^c^	453.62***
Commitment	2.93 ± 0.61^a^	2.57 ± 0.64^b^	2.01 ± 0.69^c^	416.50***
Weekly days ≥ 30 minutes PA	2.70 ± 1.07^a^	2.48 ± 1.15^b^	2.02 ± 1.22^c^	70.14***
Weekly minutes spent on:				
MVPA (total)	537 ± 575^a^	434 ± 491^b^	362 ± 506^b^	22.56***
Sports	138 ± 184^a^	90 ± 127^b^	56 ± 172^c^	52.60***
Walking during spare time	102 ± 156^a^	90 ± 159^ab^	79 ± 153^b^	4.43*
Walking to work/school	20 ± 140	18 ± 72	15 ± 67	0.47
Biking during spare time	74 ± 175^a^	62 ± 172^a^	38 ± 110^b^	9.57***
Biking to work/school	34 ± 88^a^	34 ± 97^a^	17 ± 44^b^	9.27***
Gardening	66 ± 143	51 ± 111	59 ± 138	2.77
Chores	85 ± 233	70 ± 211	68 ± 199	0.86

MVPA = moderate to vigorous physical activity.

For each variable, means with different superscripts indicate a significant difference at P < .05 using Bonferroni post-hoc tests.

*P < .05; **P < .01; ***P < .001.

### Differences between clusters: IMI subscales, intention & commitment

The MANOVA regarding the IMI subscales, intention and commitment was significant (Pillai’s trace 0.542; *p* < .001), suggesting between-cluster differences on these variables. Follow-up ANOVAs indicated between-cluster differences on all subscales of the IMI, as well as on intention and commitment (see Table 3). According to the Bonferroni post-hoc tests, compared to the other two clusters, members of the *autonomous motivation* cluster scored significantly higher on interest/enjoyment, perceived competence, effort/importance, perceived choice and value/usefulness, and significantly lower on pressure/tension. Compared to the *low motivation* cluster, members of the *controlled motivation* cluster scored significantly higher on interest/enjoyment, perceived competence, effort/importance, pressure/tension and value usefulness, and significantly lower on perceived choice. Bonferroni post-hoc tests indicated that individuals from the *autonomous motivation* cluster scored significantly higher on intention and commitment than those from the other two clusters. Members of the *controlled motivation* cluster scored significantly higher on both variables when compared to members of the *low motivation* cluster.

### Differences between clusters: PA behavior

Although the PA variables were positively skewed, no transformations were needed since the effect of non-normality on ANOVAs is rather small provided that the sample sizes are large (Zar 1996). Compared to the other two clusters, individuals from the *autonomous motivation* cluster reported significantly more weekly days with ≥ 30 minutes PA, more weekly minutes spent on total MVPA and more weekly minutes spent on sports. Furthermore, they reported more weekly minutes walking during spare time than did members of the *low motivation* cluster. Compared to members of the *low motivation* cluster, members of the *controlled motivation* cluster reported significantly more weekly days with ≥ 30 minutes PA, more weekly minutes spent on MVPA and more weekly minutes spent on sports. Individuals from the *autonomous motivation* cluster and the *controlled motivation* cluster reported significantly more weekly minutes spent on biking to work/school and biking during spare time than did those from the *low motivation* cluster.

## Discussion

The present study aimed to reveal motivational profiles based on SDT in a large sample of adults not complying with PA guidelines, and to assess the differences between the derived clusters in terms of demographics, PA level, intention, commitment and subjective experience with regard to PA. Similar to previous research (Matsumoto & Takenaka 2004; Ntoumanis 2002; Boiché et al. 2008), the present study showed that cluster analysis was able to identify groups of individuals based on motivational regulations.

Three clusters were found: (1) the *autonomous motivation* cluster *-* individuals in this cluster scored high on autonomous motivation and low to moderate on controlled motivation; (2) the *controlled motivation* cluster – individuals in this cluster scored high on controlled motivation and moderate on autonomous motivation; and (3) the *low motivation* cluster – individuals in this cluster scored low to moderate on controlled motivation and low on autonomous motivation. This cluster solution was similar to those found in earlier studies on active individuals (Matsumoto & Takenaka 2004; Ntoumanis 2002; Boiché et al. 2008). These studies also showed a cluster characterized by high autonomous motivation and a cluster with high controlled motivation (Matsumoto & Takenaka 2004; Ntoumanis 2002; Boiché et al. 2008). Compared to Guerin (Guerin & Fortier 2012) who also assessed relatively inactive adults, many similarities can be observed. In both studies, three clusters were found, of which one was characterized by high levels of autonomous motivation, and one scored low on all motivational regulations (Guerin & Fortier 2012). However, since the cluster analyses in our study used z-scores (as recommended by Hair (Hair & Black 2000; Hair et al. 1998)) and not raw scores as in Guerin (Guerin and Fortier 2012), it is hard to compare the profiles in detail. Using raw scores in order to form clusters can lead to slightly different results than when z-scores are used (Hair & Black 2000; Hair et al. 1998). Furthermore, in the study by Guerin, a different measure was used to assess motivational regulations, which also makes it more difficult to compare the results of these two studies.

In our study, the *autonomous motivation* cluster was the largest cluster (53.0%). This was not expected since all individuals in the sample reported less than 5 weekly days of ≥ 30 minutes PA. The large percentage of autonomously motivated individuals in our research population could be related to the fact that our sample consisted of individuals who agreed to participate in an intervention study. Since these individuals chose to participate in the trial, they may be on average somewhat more motivated to increase their PA level, compared to those individuals who chose not to participate in the trial (Hall et al. 2010).

In the present study, the *autonomous motivation* cluster displayed more favorable characteristics when compared to the *controlled motivation* and the *low motivation* cluster. Individuals from the *autonomous motivation* cluster spent more time on MVPA and sports and their BMI was lower than in the other two clusters. With regard to functional lifestyle activities such as active transport and chores, the differences between the *autonomous motivation* cluster and the other two clusters were less significant or even absent. These findings remind us that PA behavior is a broad concept that comprises different sub-behaviors (Marttila et al. 1998). More specifically, the findings imply that habitual lifestyle physical activities and sports should be treated as different constructs (Silva et al. 2010; Burton et al. 2006; Donnelly et al. 2009). It may well be that autonomous motivation plays a bigger role for the maintenance of sports than it does for sustaining daily lifestyle PA. For lifestyle PA, habit and pragmatic motives may be the most important driving force (de Bruijn & Gardner 2011) while for sports, intrinsic motives such as fun or challenge may be at play (Teixeira, Carraca et al. 2012). However, the present study is cross-sectional, so no causality can be inferred, and there may also be an effect in the opposite direction. For instance, individuals in the *autonomous motivation* cluster, who are in general more active than those in the other two clusters, may feel more positively towards more strenuous forms of PA simply because they have more experience with it.

In addition to being more physically active, members of the *autonomous motivation* cluster reported more favorable scores in terms of subjective experience while being physically active than members of the other clusters. As suggested by Buckworth and colleagues, endorsement of such factors is associated with continued participation in regular PA (Buckworth et al. 2007). Compared to individuals from the other two clusters, individuals from the *autonomous motivation* cluster experience more enjoyment, more free choice and less stress while being active. They also felt more confident about their PA skills, put more effort into PA and they perceive PA as more valuable for themselves. This suggests that studies on PA motivation would benefit from including measures of subjective experience while being active such as the IMI (McAuley et al. 1989). Most studies on PA motivation only assess motivational regulations (Teixeira, Carraca et al. 2012). Inclusion of variables reflecting subjective experience while being active provides valuable information since these variables show how an individual’s motivation is related to how he or she experiences PA.

Members of the *autonomous motivation* cluster also scored higher on intention and commitment towards PA than those in the other two clusters. These results indicate that a motivational profile characterized by high autonomous motivation and low controlled motivation is not only associated with a more active lifestyle, but it also offers the most promising starting point for becoming even more physically active. This interpretation was supported by the present literature on SDT and PA which shows that autonomous motivation is an important predictor of uptake and maintenance of strenuous PA (Teixeira, Carraca et al. 2012; Silva et al. 2010; Edmunds et al. 2006).

When comparing the *controlled motivation* cluster to the *low motivation* cluster, several observations can be made. In general, individuals from the *controlled motivation* cluster displayed more favorable scores than those from the *low motivation* cluster. For instance, individuals from the *controlled motivation* cluster spent more time on sports and displayed higher intention and commitment scores towards PA. Also, they experienced more interest in PA and placed more importance on being physically active. These results suggest that a motivational profile characterized by high controlled motivation and low autonomous motivation better enables PA increase than a profile with low scores on both types of motivation. Prior SDT research acknowledges that controlled motivation can sometimes be important, as it is often a predictor of intention formation during the very first steps towards an active life (Markland & Ingledew 2007). Indeed, it may be that individuals from the *controlled motivation* cluster are more likely to become more physically active than those from the *low motivation* cluster, as a consequence of controlled motives such as weight management and appearance (Markland & Ingledew 2007). Notably, the results of the present study showed that individuals from the *controlled motivation* cluster displayed less perceived choice and higher pressure/tension than individuals from the *low motivation* cluster. These feelings of pressure and obligation are probably related to the compulsory (“I should/ought to”) nature of controlled motivation (Teixeira, Silva et al. 2012; Ng et al. 2013).

The present study has several strengths. One important strength concerns the large research sample, consisting of adults not complying with PA guidelines. As mentioned before, this population is rather underrepresented in the existing literature on motivational profiles. In the current study, a variety of (PA related) self-report measures were assessed, and this enabled us to obtain a clear picture of the characteristics of the profiles as well as the between-cluster differences. Also, the cluster analysis in this study was conducted using a clear analysis protocol (Hair & Black 2000; Hair et al. 1998). Despite these strengths, the study also has some limitations. First, it should be noted that the design is cross sectional. Therefore, it is not possible to infer causal relationships from the results. Second, PA behavior was assessed using self-report. Although the reproducibility and relative validity of the measurement instrument are reasonable (Wendel-Vos et al. 2003), this should be viewed as a limitation of this study. Third, it should be underscored that the research sample consists of individuals who agreed to participate in an intervention trial. Since these individuals chose to participate in the trial, they probably already developed some motivation to become more active, which may preclude generalization of the results to the general population (Hall et al. 2010). Fourth, the present study focused on relatively inactive individuals, while in many of the SDT items in the questionnaire – such as “I enjoy being physically active” – it is assumed that the participant has some daily experience with PA. However, as only four participants reported zero weekly minutes of PA, we do not think this influenced the results of the study. Lastly, while interpreting the results of this study, it is important to note that the SRQ-E subscales “intrinsic motivation” and “identified regulation” are probably very closely related to the IMI subscales “interest/enjoyment” and “value/usefulness”, respectively. Strictly speaking, however, the SRQ-E measures aim to assess the participant’s conscious motives for being physically active, while IMI subscales simply assess the experiences that participants have while *being* active, regardless of whether that experience motivates them to *become* active.

Several implications can be drawn from this study. First, it shows that cluster analysis is a useful method for differentiating between motivational profiles in a large group of individuals who do not comply with PA guidelines. This approach provides more information about an individual’s motivation than just categorizing him or her as high or low in autonomous motivation. In addition, the results of this study provide additional support for the importance of autonomous motivation in the context of PA behavior. From this perspective, PA promotion workers should not focus on producing immediate increases in PA behavior in their clients by using external pressures (Patrick et al. 2013). Instead, by applying a client-centered counseling style, such as Motivational Interviewing (Miller & Rollnick 2013; Vansteenkiste & Sheldon 2006), practitioners can support their clients’ basic psychological needs, and help them to develop autonomous motivation (Patrick et al. 2013). At the same time, clients in PA (counseling) interventions might benefit from slightly different intervention approaches, depending on their motivational profile. Individuals with overall low motivation might benefit most from exploring personally relevant reasons for becoming active. Individuals who are mainly driven by controlled motivation may profit most from exploring goals and developing PA plans that better suit their core values (Miller & Rollnick 2013). Those who are already autonomously motivated, may be helped by an approach that reinforces their intrinsic motives, and helps them to develop more challenging PA plans.

## Conclusions

In the present study three motivational clusters were derived: a *low motivation* cluster, a *controlled motivation* cluster and an *autonomous motivation* cluster. These clusters differed significantly from each other with respect to PA behavior, motivation to be active and subjective experience while being active. The results show that the combination of high autonomous motivation and low controlled motivation is most associated with an active lifestyle and with beneficial scores on the PA-related psychological measures.

The results of this study provide additional support for the importance of autonomous motivation in the context of PA behavior. The three derived clusters may be relevant in the context of PA interventions as individuals with different motivational profiles might benefit most from different intervention approaches. Finally, this study shows that cluster analysis is a useful method for differentiating between motivational profiles in large groups of individuals who do not comply with PA guidelines.

## Abbreviations

ANOVA: Analysis of variance
IMI: Intrinsic motivation inventory
MANOVA: Multivariate analysis of variance
MVPA: Moderate to vigorous physical activity
PA: Physical activity
SDT: Self-determination theory
SRQ-E: Exercise self-regulation questionnaire
SQUASH: Short questionnaire to assess health enhancing physical activity
